# Screening of Six Medicinal Plant Extracts Obtained by Two Conventional Methods and Supercritical CO_2_ Extraction Targeted on Coumarin Content, 2,2-Diphenyl-1-picrylhydrazyl Radical Scavenging Capacity and Total Phenols Content

**DOI:** 10.3390/molecules22030348

**Published:** 2017-02-24

**Authors:** Maja Molnar, Igor Jerković, Dragica Suknović, Blanka Bilić Rajs, Krunoslav Aladić, Drago Šubarić, Stela Jokić

**Affiliations:** 1 Josip Juraj Strossmayer University of Osijek, Faculty of Food Technology Osijek, Franje Kuhača 20, 31000 Osijek, Croatia; mmolnar@ptfos.hr (M.M.); bbilic@ptfos.hr (B.B.R.); drago.subaric@ptfos.hr (D.Š.); stela.jokic@ptfos.hr (S.J.); 2 Faculty of Chemistry and Technology, University of Split, R. Boškovića 35, 21000 Split, Croatia; 3 Department of Clinical Laboratory Diagnostics, University Hospital Centre Osijek, Huttlerova 4, 31000 Osijek, Croatia; dragica.suknovic@gmail.com; 4 Croatian Veterinary Institute, Branch, Veterinary Institute Vinkovci, Josipa Kozarca 24, 32100 Vinkovci, Croatia; k2aladic@gmail.com

**Keywords:** medicinal plants, extraction, supercritical CO_2_ extraction, coumarin, antiradical capacity

## Abstract

Six medicinal plants *Helichrysum italicum* (Roth) G. Don, *Angelica archangelica* L., *Lavandula officinalis* L., *Salvia officinalis* L., *Melilotus officinalis* L., and *Ruta graveolens* L. were used. The aim of the study was to compare their extracts obtained by Soxhlet (hexane) extraction, maceration with ethanol (EtOH), and supercritical CO_2_ extraction (SC-CO_2_) targeted on coumarin content (by high performance liquid chromatography with ultraviolet detection, HPLC-UV), 2,2-diphenyl-1-picrylhydrazyl radical (DPPH) scavenging capacity, and total phenols (TPs) content (by Folin–Ciocalteu assay). The highest extraction yields were obtained by EtOH, followed by hexane and SC-CO_2_. The highest coumarin content (316.37 mg/100 g) was found in *M. officinalis* EtOH extracts, but its SC-CO_2_ extraction yield was very low for further investigation. Coumarin was also found in SC-CO_2_ extracts of *S. officinalis*, *R. graveolens*, *A. archangelica*, and *L. officinalis*. EtOH extracts of all plants exhibited the highest DPPH scavenging capacity. SC-CO_2_ extracts exhibited antiradical capacity similar to hexane extracts, while *S. officinalis* SC-CO_2_ extracts were the most potent (95.7%). EtOH extracts contained the most TPs (up to 132.1 mg gallic acid equivalents (GAE)/g from *H. italicum*) in comparison to hexane or SC-CO_2_ extracts. TPs content was highly correlated to the DPPH scavenging capacity of the extracts. The results indicate that for comprehensive screening of different medicinal plants, various extraction techniques should be used in order to get a better insight into their components content or antiradical capacity.

## 1. Introduction

Medicinal plants are rich in bioactive compounds with protective and healing properties that are often present in low concentrations. Since they are often chemically sensitive, it is very important to select an appropriate method for their isolation, purification, and quantification.

As one of the bioactive plant compounds, coumarins form a large class of plant secondary metabolites. Coumarin (2*H*-chromen-2-one or 1-benzopyran-2-one) and its derivatives are considered phenylpropanoids biosynthesized from shikimic acid-derived phenylpropane precursors. Some naturally occurring coumarin derivatives include umbelliferone (7-hydroxycoumarin), aesculetin (6,7-dihydroxycoumarin), herniarin (7-methoxycoumarin), psoralen, and imperatorin. Coumarins are widely distributed in various plant families, such as Apiaceae, Asteraceae, Fabiaceae, Rosaceae, Rubiaceae, Solanaceae, especially Rutaceae and Umbelliferae [1,2,3,4]. They can be distributed in all plant parts depending on the growing conditions [3]; they act as phytoalexins, and are biosynthesized when the plant is subjected to adverse conditions like wilting, disease, or drought. Their protective role in the plants is also expressed as antifungals and insect repellents [4,5,6]. Coumarin classification in different groups is based on their structural differences and depending on which they show a wide range of pharmacological effects, such as antiinflammatory effect in vitro and in vivo, analgesic effect [7,8,9], antimicrobial activity [7,10,11], lipid peroxidation inhibition [12,13], and others. Coumarins are known for their allelopathic activities [2,14], including antibacterial, nematocidal, and insecticidal activities, as well as phytotoxic activity on other plants [15]. Coumarins have found a wide range of applications, particularly in cosmetic and pharmaceutical industries [16]. Various extraction techniques have been employed for the isolation of coumarins from various plant materials, such as maceration, ultrasound maceration, or infusion with aqueous ethanol, water, methanol, ethyl acetate, chloroform, diethyl ether, or other solvents [17,18,19,20].

For the present research, six commercially available medicinal plants often used in Croatia were selected: *Melilotus officinalis* L., *Ruta graveolens* L., *Angelica archangelica* L., *Salvia officinalis* L., *Lavandula officinalis* L., and *Helichrysum italicum* G. Don. Only specific parts of each plant were chosen based upon their common usage, mostly in tea preparations and tinctures. *H. italicum* flower preparations have found different medicinal uses; e.g., for toothache, digestive disorders, wound healing, intestinal parasitic infections, asthma, etc. [21]. *Angelica* roots are used in traditional medicine as well as spice [22], lavender flowers in phytotherapy [23], and yellow melilot herb exhibits well-known medicinal uses and is included in the European Medicines Agency catalogue [24]. The incorporation of such plant materials or their extracts in different foodstuffs and the increasing demand for naturally derived seasonings, cosmetics, and dietary supplements requires a screening of the compounds whose limits are regulated by law. Coumarin content in different foodstuffs and cosmetic products is limited by European legislation—EC regulation 1334/2008 [25]. Detailed insight into the coumarin content in selected plants obtained by different methods, particularly by supercritical CO_2_ (SC-CO_2_) extraction, is missing. *M. officinalis* is a well-known coumarin containing plant investigated by Martino et al. [26], who noticed that the applied extraction conditions exhibited a great influence on coumarin concentration. *R. graveolens* contains numerous coumarin compounds, as well as coumarin itself [27]. Stashenko et al. [28] applied subcritical CO_2_ extraction on the flowers, leaves, stems, and roots of *R. graveolens*, and the highest coumarin concentration was found in the roots. *A. archangelica* is also a well-known coumarin-containing plant, and aside from coumarin derivatives being determined in this plant [29,30,31], data on coumarin content itself are lacking in the literature. Very popular Mediterranean plants in Croatia, namely sage (*S. officinalis*), lavender (*Lavandula* sp.), and immortelle (*H. italicum*) were also investigated. Comprehensive research of different extraction techniques on the selected six plants presents novelty, particularly regarding green chemistry area focused on more resource-efficient and inherently safer design of extraction targeted to coumarin content, 2,2-diphenyl-1-picrylhydrazyl radical (DPPH) scavenging capacity, and total phenols (TPs) content. The goals of present research are to (a) obtain the extracts from selected plants by various techniques (Soxhlet extraction with hexane, maceration with 96% and 50% ethanol, and SC-CO_2_ extraction at 300 bar and 150 bar for selected samples); (b) determine and compare the extraction yields for the same plant among various methods and different extraction conditions; (c) analyze coumarin amount by high performance liquid chromatography with ultraviolet detection (HPLC-UV) and compare it within the extracts of the same plant as well as different samples; (d) investigate DPPH scavenging capacity of the obtained extracts; (e) determine TPs content in all extracts by spectroscopic Folin–Ciocalteu assay and correlate it with measured DPPH scavenging capacity.

## 2. Results and Discussion

The selected extraction techniques were ubiquitous Soxhlet extraction with hexane, maceration with EtOH, and SC-CO_2_ extraction (as a green technique and a good alternative to conventional organic solvents). SC-CO_2_ and hexane possess similar dissolving capacity. EtOH as a polar solvent was also chosen for maceration, since it is often used in herbal pharmacy for the production of tinctures [32]. The research was designed to compare the extracts obtained by SC-SO_2_ with hexane extracts (solvents with similar polarity), and to compare the extracts obtained by SC-SO_2_ with EtOH extracts (solvents with different polarity) with respect to the extraction yields, coumarin content, DPPH scavenging capacity, and TPs content of the obtained extracts.

Comparing the extraction yield of the applied extraction techniques, the same pattern for all plants can be observed (Table 1). The extraction yield was expressed as % (g of extract/100 g of dried plant material), and the obtained extracts were of oily composition or solids, so they were weighted for all analytical assays. The highest yields were obtained using EtOH as the solvent (maceration), followed by hexane extraction, and last SC-CO_2_ extraction.

The highest extraction yield of 14.95% was obtained using 50% EtOH from *R. graveolens*. In all samples, the highest yields were obtained with 50% EtOH rather than 96% EtOH. In general, EtOH extraction provided much higher yields in comparison with other used solvents (Table 1).

The extraction yields obtained with SC-CO_2_ were comparable to the yields obtained with hexane. This can be explained by the similar dissolving capacity of SC-CO_2_ and hexane (both are non-polar solvents dissolving non-polar compounds), while EtOH as polar solvent dissolved polar compounds. However, supercritical fluid extraction (SFE) is an attractive alternative to the other methods used because of the possibility of producing plant extracts without any trace of conventional organic solvents, and which are thus directly usable. When comparing SC-CO_2_ extraction yields applying two different pressures (150 bar and 300 bar), several differences can be seen among the plants. SC-CO_2_ extraction yield for *S. officinalis* is higher at 300 bar than at 150 bar, which is in accordance with the data published by Glisic et al. [33]; the yield increased from 0.92% for the extraction at 70 bar and 50 °C to 4.82% for SC-CO_2_ extraction at 300 bar. For *R. graveolens*, *M. officinalis*, and *A. archangelica*, SC-CO_2_ extraction yields were very low—especially at 150 bar; therefore, coumarin content, antiradical capacity, and TPs content of those extracts were not determined.

**Figure 1:**
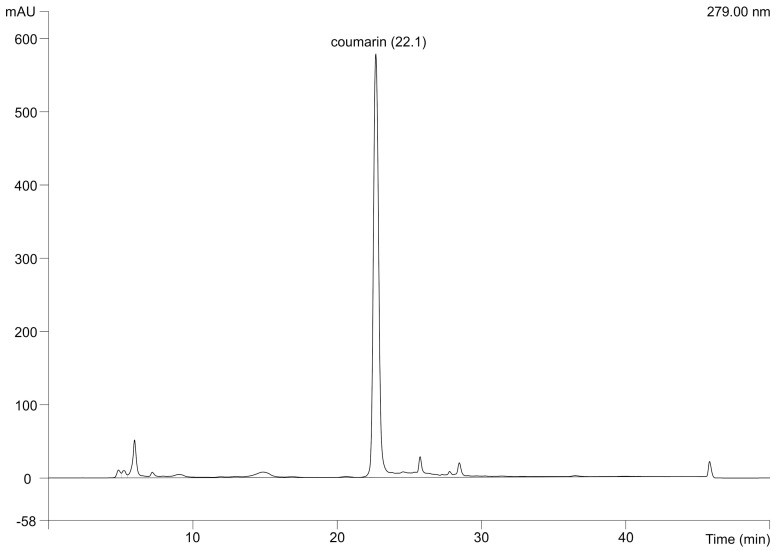
The chromatogram of *M. officinalis* L. extract obtained with 96% EtOH (mAU: milli absorbance unit).

The highest coumarin content was found in *M. officinalis* (Table 2), especially in the 96% EtOH extract (316.4 mg/100 g) followed by the 50% EtOH extract (146.4 mg/100 g) and hexane extract (8.9 mg/100 g). The HPLC chromatogram of *M. officinalis* 96% EtOH extract with the highest coumarin content is given in Figure 1.

*M. officinalis* is well known as coumarin-containing plant, and other authors have investigated the content of coumarin in the extracts gained by different techniques or applying different extraction solvents. Wu et al. [15] showed that after extraction using organic solvents with different polarity (petroleum ether, ethyl acetate, and butanol) the highest coumarin concentration was obtained with petroleum ether. Martino et al. [26] investigated Soxhlet extraction with 95% EtOH, ultrasound-assisted extraction (USAE) with 50% EtOH, and microwaves-assisted extraction (MAE) with 50% EtOH of *M. officinalis* flowering tops in closed system and determined the content of coumarin by USAE (1.19–3.62 mg/g), by MAE (2.44–3.98 mg/g), and by Soxhlet extraction (2.15 mg/g).

For *S. officinalis*, coumarin was detected in SC-CO_2_ extracts, with higher yield at 150 bar than 300 bar, while it was not found in EtOH and hexane extracts. This is the first time that coumarin has been reported in extract of *S. officinalis*.

*R. graveolens* contains coumarin derivatives (marmesin, scopoletin, isopimpinellin, hydroxyl-coumarin, xanthotoxin, umbelliferone, isoimperatorin, psoralen, bergapten, and herniarin [34]), but it also contains coumarin itself [7,27]. In the present research, the coumarin content in *R. graveolens* was 0.47 mg/100 g for hexane extracts and 0.53 mg/100 g for SC-CO_2_ (300 bar) extracts.

*A. archangelica* contains coumarin derivatives (isoimperatorin, oxypeucedanin, imperatorin, ostruthol, angelicin, bergapten, scopoletin, isopimpinellin, and xanthotoxin [29,30]), but limited data are available on its coumarin content, a biosynthetic precursor of the mentioned derivatives. In the present study it was found that *A. archangelica* contains coumarin (0.91 mg/100 g) when SC-CO_2_ extraction is performed under 300 bar.

Coumarin content in *L. officinalis* extracts was similar using 96% EtOH and SC-CO_2_ as the solvents, while coumarin was not found in the extracts obtained with 50% EtOH and hexane. Our results are comparable to those of Areias et al. [23], who extracted phenolic compounds from lavender flowers and found that the content of coumarin was 0.7–2.63 mg/100 g (dry basis).

Although *H. italicum* is also known to contain coumarins, coumarin itself was not found in any of the extracts. However, scopoletin was identified in our previous work [35]. Upon the application of SC-CO_2_ extraction parameters on *H. italicum* flowers, scopoletin yield varied from 0.024 mg/100 g to 1.933 mg/100 g depending on SFE operating conditions.

Antiradical capacity of all extracts was expressed as DPPH scavenging activity and determined at the same concentration (250 μg/mL) for all extracts (Table 3). Several extracts have shown high antiradical capacity (i.e., 100%) at this concentration, so IC_50_ was determined for those extracts. In general, EtOH extracts of all investigated plants showed higher DPPH scavenging activity than the ones obtained with other solvents. *S. officinalis* extracts were found to possess an excellent antioxidant activity, with IC_50_ = 25.9 μg/mL and 32.49 μg/mL for hexane and 96% EtOH extracts. SC-CO_2_ extracts (300 bar and 150 bar) of *S. officinalis* also showed very high antiradical capacity, with IC_50_ = 79.8 μg/mL and 160.27 μg/mL the highest among all investigated plants. Many researchers have investigated the antioxidant activity of *S. officinalis* extracts, claiming that it was related to the major marker compounds carnosic acid, carnosol, and rosmarinic acid, as well as flavonoids and other phenolics [36,37]. EtOH extract of *R. graveolens* also showed a great antiradical capacity, with IC_50_ = 89.5 μg/mL, as well as *H. italicum* hexane and EtOH extracts with IC_50_ = 52.1 and 44.5 μg/mL, respectively.

In general, there is no correlation between coumarin content and antiradical activity, since coumarin itself does not scavenge DPPH radicals probably due to its limited ability to delocalize electrons. Its measured antiradical activity expressed as DPPH scavenging activity was only 1.08%. However, it is well known from different phytochemical studies that plants flavonoids and other polyphenolic compounds could be responsible for the antiradical activity (e.g., as in genus *Salvia*), and the content of phenolics in the extracts correlates with their antiradical activity [38]. Therefore, total phenols (TPs) content was determined in all obtained extracts by spectroscopic Folin–Ciocalteu assay (Table 4). In general, EtOH extracts exhibited higher TPs content in comparison with hexane or SC-CO_2_ extracts, probably due to the high polarity of the extraction solvent. TPs content was the highest in the extracts from *H. italicum* (132.1 mg gallic acid equivalent (GAE)/g) and *S. officinalis* (90.6 mg GAE/g). SC-CO_2_ extracts of those two plants also contained the highest TPs content (Table 4) in comparison with other samples.

When TPs content was compared to the antiradical activity data, a high correlation of 87% was obtained, indicating that phenols greatly influence the antiradical activity of all extracts. The highest TPs content was determined in *H. italicum* and *S. officinalis* EtOH extracts, both possessing an excellent antiradical activity.

## 3. Materials and Methods

### 3.1. Chemicals

The purity of CO_2_ used for the extraction was 99.97% (*w*/*w*) (Messer, Osijek, Croatia). DPPH and ethyl acetate were purchased from Sigma-Aldrich Chemie (Steinheim, Germany). Coumarin standard was purchased from Dr. Ehrenstorfer GmbH (Augsburg, Germany), and standard purity was 99.5%. All solvents were of analytical grade and purchased from J.T. Baker (Center Valley, PA, USA).

### 3.2. Plant Material

Six medicinal plants (Table 5) were used in this study for the production of different extracts. Immortelle flowers were collected from a plantation of ca. 14 ha from Ljubuski, Herzegovina region (Bosnia and Herzegovina) at the beginning of July 2015 and then air-dried in the shade for several days. Other plants angelica, lavender, sage, yellow melilot and rue were purchased from herbal pharmacy Vextra d.o.o. (Mostar, Bosnia, and Herzegovina) in the spring of 2015. Before the extraction, the plant material was ground using a laboratory mill.

### 3.3. Determination of Initial Water Content

The moisture content of the plant materials was determined according to the Association of Official Analytical Chemists (AOAC) Official Method [39]. The measurement was done in triplicate.

### 3.4. Soxhlet Extraction

A sample of 5.0 g of each plant material was extracted with 150 mL *n*-hexane using a Soxhlet apparatus for 8 h. Subsequently, the solvent was evaporated under vacuum, and the obtained extract was stored in a glass bottle at 4–6 °C. Triplicate extractions were performed.

### 3.5. Alcoholic Extracts Processing

The 20.0 g of dried and ground material were immersed into 100 mL of 96% EtOH. The same mass of plant material was also put in 50% ethanol. The systems were left to soak for 5 days in the dark at room temperature and were occasionally shaken. The alcoholic extract was then filtered through filter paper to eliminate any solid impurity and concentrated in a rotary vacuum evaporator at 35 °C, yielding a waxy material. Finally, the extracts were kept in the dark at 4–6 °C until tested. Triplicate extractions were performed.

### 3.6. Supercritical CO_2_ Extraction

The experiment was performed in a supercritical fluid extraction system explained in detail previously [40]. The dried and ground material of each medicinal plant (100.0 g) was placed into the extractor vessel, and the extracts were collected in a separator in glass tubes at 15 bar and 25 °C. The extraction was performed at an extraction pressure of 300 bar, a temperature of 40 °C, and a CO_2_ mass flow rate of 1.95 kg/h. The same experiment was also performed at 150 bar, but the extraction yields were very low for some plants, as indicated in Table 1. The mass of dried material in the extractor, the extraction time, and CO_2_ mass flow rate were kept constant during experiments. CO_2_ flow rate (2 kg/h) was measured by a Matheson FM-1050 (E800) flow meter (Matheson Tri-Gas, Inc., Basking Ridge, NJ, USA). Each extraction run lasted for 90 min, since longer extraction times did not significantly increase the extraction yield (based on our preliminary experiments). The extracts were kept at 4–6 °C until HPLC analyses. Triplicate extractions were performed.

### 3.7. Determination of Coumarin Concentration by High Performance Liquid Chromatography

Determination of coumarin in the obtained extracts was performed using reverse phase (RP)-HPLC method with UV detection. The analysis was performed on a Varian ProStar system (Varian Analytical Instruments, Palo Alto, CA, USA) containing Varian ProStar 230 Solvent Delivery Module, ProStar 500 Column Valve Module (Varian Analytical Instruments), and ProStar 330 Photodiode Array detector (Varian Analytical Instruments) and coupled to a computer with the ProStar 5.5 Star Chromatography Workstation and PolyView 2000 V 6.0. (Varian Analytical Instruments). COSMOSIL 5C18-MA-II (NacalaiTesque, Inc., Kyoto, Japan) column, 150 mm long with internal diameter of 4.6 mm was used for chromatographic separation. Gradient elution with distilled water as phase A and methanol as phase B was used for separation, with the following gradient: 0–15 min, 60% A and 40% B phase; 15–20 min, increasing the share of phase B to 80% and decreasing phase A to 20%; 20–40 min, holding 20% A and 80% B phase; 40–41 min decreasing of B phase to 40% and increasing A phase to 60%, 41–50 min, holding 60% A and 40% B phase. The analyses were performed at room temperature, with flow rate 1.0 mL/min, injection volume 20 µL, and UV detection wavelength 279 nm. The stock solutions of coumarin standard were prepared in a solvent, and calibration was obtained at six concentrations (concentration range 1.0, 2.0, 5.0, 10.0, 20.0, 30.0 mg/L). Linearity of the coumarin calibration curve was confirmed by R^2^ = 0.9997. Coumarin limit of detection (LOD) was 0.035 mg/L, limit of quantification (LOQ) 0.345 mg/L, and compound retention time was 22.1 min. The extracts were weighted, diluted in HPLC grade methanol, filtered through 0.45 μm polytetrafluoroethylene (PTFE) filters, and subjected to HPLC analyses. Coumarin concentration in the plant extracts (μg/mL) determined by HPLC analysis (in triplicate) was recalculated to mg of coumarin/100 g of the plant sample.

### 3.8. Determination of DPPH Antiradical Capacity

Antiradical activity of the obtained extracts was determined using the DPPH method described earlier [41]. The plant extracts were dissolved in ethyl acetate (250 μg/mL) and mixed with 0.3 mM DPPH radical solution. Determination of coumarin antiradical activity was performed as described in our previous paper [42], using methanol as solvent. All measurements were done in triplicate. The absorbance was measured at 517 nm, and DPPH scavenging activity was determined using Equation (1):

(1) $$DPPH\;\mathrm{acticity}\;(\%)=\frac{(A_{\mathrm{DPPH}}+A_{s})-A_{s}}{A_{\mathrm{DPPH}}}*100$$

### 3.9. Determination of Total Phenolics Content

Total phenolics content of the extracts was determined by a modified spectrophotometric method with Folin-Ciocalteu reagent, calibrated against gallic acid [43]. The results were calculated according to the calibration curves for gallic acid and TPs mass fraction, derived from triplicate analyses and expressed as mg of gallic acid equivalents (GAE) per g of the extracts. The correlation analysis among TPs content and DPPH scavenging capacity was performed using Statistica 8.0 software (Stat Soft Inc., Tulsa, OK, USA).

## 4. Conclusions

This study provides insight into different extraction techniques and conditions for the preparation of medicinal plant extracts considering extraction yield, coumarin content and antiradical capacity. The highest extraction yields were obtained using EtOH, followed by hexane and SC-CO_2_ extractions. As expected, the highest coumarin content was found in EtOH extracts of *M. officinalis*, but its SC-CO_2_ extraction yield was too low for further investigation. However, coumarin was found in SC-CO_2_ extracts of *S. officinalis* (first time report), *R. graveolens*, *A. archangelica*, and *L. officinalis*. Therefore, SC-CO_2_ could be interesting for the extraction of other constituents containing coumarin as building block with substituents of varying complexity. EtOH extracts of all plants showed the highest DPPH scavenging activity. However, all SC-CO_2_ extracts exhibited antiradical capacity similar to hexane extracts, while SC-CO_2_ extracts of *S. officinalis* were the most potent. However, great variability among obtained SC-CO_2_ extraction yields from six medicinal plants in comparison with other applied methods indicates that different extraction techniques should be used for comprehensive screening.

## Figures and Tables

**Table 1:** The percentages of extraction yields and moisture content of the plant materials.

Properties	*H. italicum*	*A. archangelica*	*L. officinalis*	*S. officinalis*	*M. officinalis*	*R. graveolens*
Moisture content (%)	12.88 ± 0.01	12.02 ± 0.04	11.93 ± 0.01	12.42 ± 0.06	13.66 ± 0.04	12.19 ± 0.08
Soxhlet extraction	4.95 ± 0.24	2.39 ± 0.29	4.13 ± 0.18	5.33 ± 0.38	1.29 ± 0.04	2.03 ± 0.27
96% EtOH	6.70 ± 0.28	6.15 ± 0.41	9.75 ± 0.33	9.50 ± 0.22	4.40 ± 0.04	9.95 ± 0.34
50% EtOH	10.1 ± 0.36	11.75 ± 0.48	12.3 ± 0.29	10.35 ± 0.48	10.00 ± 0.49	14.95 ± 0.44
SC-CO_2_ (300 bar)	4.85 ± 0.20	0.35 ± 0.11	2.19 ± 0.31	4.28 ± 0.31	0.05 ± 0.03	0.60 ± 0.11
SC-CO_2_ (150 bar)	2.86 ± 0.56	<0.01	2.65 ± 0.51	3.77 ± 0.19	<0.01	<0.01

*H. italicum*: *Helichrysum italicum* G. Don.; *A. archangelica*: *Angelica archangelica* L.; *L. officinalis*: *Lavandula officinalis* L.; *S. officinalis*: *Salvia officinalis* L.; *M. officinalis*: *Melilotus officinalis* L.; *R. graveolens*: *Ruta graveolens* L.; SC-CO_2_: supercritical CO_2_.

**Table 2:** Coumarin concentration (mg/100 g) in the plant extracts.

Extraction Method	*H. italicum*	*A. archangelica*	*L. officinalis*	*S. officinalis*	*M. officinalis*	*R. graveolens*
Hexane extraction	0.00	0.00	0.00	0.00	8.86 ± 0.67	0.47 ± 0.11
Extraction with 96% EtOH	0.00	0.00	3.77 ± 0.61	0.00	316.37 ± 8.10	0.00
Extraction with 50% EtOH	0.00	0.00	0.00	0.00	146.43 ± 9.15	0.00
SC-CO_2_ extraction (300 bar)	0.00	0.91 ± 0.09	2.92 ± 0.17	1.45 ± 0.18	n.d.	0.53 ± 0.00
SC-CO_2_ extraction (150 bar)	0.00	n.d.	3.13 ± 0.13	2.62 ± 0.00	n.d	n.d.

n.d.: not determined.

**Table 3:** Antiradical activity of the plant extracts (250 μg/mL) as % 2,2-diphenyl-1-picrylhydrazyl radical (DPPH) scavenging activity.

Extraction Method	% DPPH Scavenging Activity					
	*H. italicum*	*A. archangelica*	*L. officinalis*	*S. officinalis*	*M. officinalis*	*R. graveolens*
Hexane extraction	94.3 ± 0.06	9.5 ± 0.52	4.0 ± 1.99	100 ± 0.00	9.0 ± 0.28	16.8 ± 1.46
Extraction with 96% EtOH	93.5 ± 0.12	8.8 ± 0.31	33.2 ± 0.45	95.2 ± 0.05	35.6 ± 0.65	59.3 ± 0.61
Extraction with 50% EtOH	93.0 ± 0.17	9.0 ± 0.12	24.2 ± 0.32	93.2 ± 0.09	30.2 ± 0.98	60.3 ± 0.14
SC-CO_2_ (300 bar)	79.12 ± 0.45	1.7 ± 0.8	3.2 ± 0.65	95.7 ± 0.44	n.d.	16.8 ± 0.84
SC-CO_2_ (150 bar)	n.d.	n.d.	10.8 ± 0.92	95.3 ± 0.51	n.d.	n.d.

**Table 4:** Total phenols content in the plant extracts expressed as mg gallic acid equivalent (GAE)/g of the extract.

Extraction Method	Total Phenols (TPs)					
	*H. italicum*	*A. archangelica*	*L. officinalis*	*S. officinalis*	*M. officinalis*	*R. graveolens*
Hexane extraction	67.4 ± 3.1	17.3 ± 1.6	7.2 ± 0.0	82.3 ± 7.4	10.5 ± 0.6	11.9 ± 0.7
Extraction with 96% EtOH	132.1 ± 3.8	14.3 ± 0.4	63.4 ± 1.2	88.2 ± 7.6	47.1 ± 3.6	89.5 ± 7.0
Extraction with 50% EtOH	104.8 ± 6.0	11.8 ± 0.5	61.9 ± 2.8	90.6 ± 5.9	46.3 ± 7.4	56.6 ± 4.3
SC-CO_2_ (300 bar)	65.3 ± 0.5	8.7 ± 0.6	6.4 ± 3.9	61.8 ± 4.4	n.d.	13.3 ± 1.3
SC-CO_2_ (150 bar)	n.d.	n.d.	4.8 ± 0.3	53.8 ± 2.7	n.d	n.d.

**Table 5:** The characteristics of medicinal plants used in this study.

Common Name of Plant	Latin Name of Plant	Part of Plant
Immortelle	*Helichrysum italicum* (Roth) G. Don	flowers
Angelica	*Angelica archangelica* L.	root
Lavender	*Lavandula officinalis* L.	flowers
Sage	*Salvia officinalis* L.	leaves
Yellow melilot	*Melilotus officinalis* L.	herb
Rue	*Ruta graveolens* L.	leaves
